# Activity, Plan, and Goal Recognition: A Review

**DOI:** 10.3389/frobt.2021.643010

**Published:** 2021-05-10

**Authors:** Franz A. Van-Horenbeke, Angelika Peer

**Affiliations:** Human-Centered Technologies and Machine Intelligence Lab, Faculty of Science and Technology, Free University of Bozen-Bolzano, Bolzano, Italy

**Keywords:** activity recognition, behavior recognition, action recognition, plan recognition, goal recognition, human-computer interaction, human-machine interaction, human-robot interaction

## Abstract

Recognizing the actions, plans, and goals of a person in an unconstrained environment is a key feature that future robotic systems will need in order to achieve a natural human-machine interaction. Indeed, we humans are constantly understanding and predicting the actions and goals of others, which allows us to interact in intuitive and safe ways. While action and plan recognition are tasks that humans perform naturally and with little effort, they are still an unresolved problem from the point of view of artificial intelligence. The immense variety of possible actions and plans that may be encountered in an unconstrained environment makes current approaches be far from human-like performance. In addition, while very different types of algorithms have been proposed to tackle the problem of activity, plan, and goal (intention) recognition, these tend to focus in only one part of the problem (e.g., action recognition), and techniques that address the problem as a whole have been not so thoroughly explored. This review is meant to provide a general view of the problem of activity, plan, and goal recognition as a whole. It presents a description of the problem, both from the human perspective and from the computational perspective, and proposes a classification of the main types of approaches that have been proposed to address it (logic-based, classical machine learning, deep learning, and brain-inspired), together with a description and comparison of the classes. This general view of the problem can help on the identification of research gaps, and may also provide inspiration for the development of new approaches that address the problem in a unified way.

## 1. Introduction

The ability to recognize human actions, plans and goals is a necessary skill that future robotic systems will need to implement in order to achieve natural and intuitive human-machine interaction. In fact, this is a task that we humans perform constantly when we interact with or observe other humans. However, we do it in such a natural and effortless way that we are usually not aware of how necessary it is for our daily-life activities. For instance, if we see someone doing the dishes, we understand what that person is doing by simply observing the body movements and the context in which the action is taking place (e.g., the place, the objects involved.). In addition, we are able to guess what the person is trying to achieve, and what will be the next actions to reach that goal (e.g., after washing the dishes, the person will start to rinse them). This way, this ability allows us to understand the intentions of the observed person, as well as to predict future actions. This information, in turn, allows us to take better-grounded decisions (e.g., help the person complete the task). While the example just exposed is a very simple one, the number of possible actions and intentions that a person may take is actually countless and of very different nature, and the usefulness of this ability in our daily life is immeasurable (e.g., when we collaborate with others, when we move in crowded places.). Thus, we can consider the ability to recognize others' actions and intentions a key skill to achieve natural human-human interaction.

Having said that, it is apparent that computational systems designed to accomplish natural and intuitive human-machine interaction will need to be equipped with modules that allow them to recognize human actions and goals with a close-to-human performance. Indeed, humans find more intuitive those interactions that they are accustomed to. Research has shown that people respond to virtual agents or human-like robots in social ways and tend to interact with them similar to how they do with people (Sproull et al., 1996). Therefore, mimicking human-human interaction seems a good approach to achieve natural human-machine interaction. On the other hand, this capacity not only makes the interactions with the system more natural, it also makes the system more prepared for different tasks with humans in the loop, allows it to model and predict human behavior, and can contribute to safety (Akkaladevi and Heindl, 2015).

However, while we humans perform these tasks naturally and effortlessly, this happens because we are indeed very effective at it, and computational systems are still far from human-like performance, especially for the case of real unconstrained environments. This occurs due to several reasons that make it a complex problem to cope with. One of the issues that arises when dealing with this problem in a real unconstrained environment is the immense variety of possible actions and plans that may be encountered. As we said before, the number of actions and intentions that we are able to perform is countless, and we humans are still quite good at recognizing them. Furthermore, we are very good at understanding and learning actions and plans that we have never seen before. Another issue to consider is the fact that many different actions and plans may seem almost identical on what the performed movements are, while having completely different semantic meanings (e.g., lifting your leg in a ballet performance or in a fight). At the same time, different instances of the same action or plan may look completely different from each other in terms of the movements performed, while having the same semantic meaning (e.g., standing on tiptoe or getting on a chair to reach a high object). Different people perform the same activity in different ways, and even the same person may perform it in very different ways at different times. The development of an effective algorithm that is able to cope with these and other challenges at a close-to-human performance level seems critical if we want to achieve the desired natural human-machine interaction.

Much effort has been done in the field of activity, plan and goal recognition to deal with these issues, and many systems have been developed for very different applications, from surveillance to video games. However, most of these systems address only a part of the problem (e.g., activity recognition, plan recognition, etc.). Since these sub-problems can be considered components of the larger problem, in this review we try to provide an overview of the problem as a whole. This holistic view may inspire the development of new solutions that address the problem in a unified way.

This article reviews and surveys the literature around the topic of activity, plan, and goal recognition, and it is organized as follows: section 2 presents an overview of the main mechanisms that are believed to be behind this ability in humans. Section 3 defines the main concepts involved in the problem. Section 4 presents and compares the main types of approaches that have been proposed to address it. Finally, section 5 discusses on this literature study, the challenges that are still to be faced and possible future directions.

## 2. Action, Plan, and Intention Recognition in Humans

The ability to attribute mental states to others is what philosophers and psychologists call **theory of mind** (Premack and Woodruff, 1978). The term refers to our presumption that others have a mind, as we do not have direct access to it and we just infer its existence from observations (Premack and Woodruff, 1978). This ability allows us, first, to understand that other people's thoughts may be different from ours, and second, to think about what others (and also ourselves) are thinking (Schaafsma et al., 2015), including emotions, desires, intentions, beliefs, and knowledge. While it is related to the concept of empathy, it is not the same: Empathy refers to emotional perspective-taking, while theory of mind concerns cognitive perspective-taking (Hynes et al., 2006). This ability contributes to social skills, such as engaging in meaningful conversations, resolving conflicts, maintaining intimacy in friendships, and being more socially competent in general (Wilde Astington, 2003). In particular, it allows us to understand why someone acts in a certain way and to predict how someone will act (Kloo et al., 2010). In fact, several research studies have shown that humans attribute plans and goals to observed agents performing sequences of actions, and are able to predict the next actions (Schmidt et al., 1978; Cohen et al., 1981). All these skills also seem to contribute to executive function (which is responsible for the cognitive control of behavior), and this contribution seems to be bidirectional. This way, social competence has been shown to take part in the development of executive function (Bierman et al., 2009), and vice versa (Alduncin et al., 2014), with children with lower levels of social competence showing deficits in executive function. An interesting fact about the theory of mind is that children start developing it at around age 3–4 (Kloo et al., 2010), and therefore younger children are unable to understand that other people's beliefs or knowledge may be different from their own (Wellman and Liu, 2004). Then, these abilities continue being developed along adolescence and into adulthood.

There are mainly two theories that try to describe how this theory of mind works: the theory-theory and the simulation theory. The **theory-theory** states that humans hold a basic theory of psychology that allows them to infer the mental states of others (Ratcliffe, 2006). This way, children develop this ability by observing the world, gathering data, and revising their theories or beliefs accordingly (Scholl and Leslie, 1999), allowing them to better understand the intentions of others and predict their behavior. A detailed model that tries to describe the mechanisms behind this theory is the BDI (belief-desire-intention) model (Bratman, 1987) (which has been used in the development of intelligent software agents).

Regarding **simulation theory**, it states that we infer the intentions and future actions of others by putting ourselves in their place and simulating their cognition, using our mind as a model of theirs (Gordon, 1986). This way, this inference implies activating mental states that, if carried into action, they would produce a similar behavior to the one observed. This explanation has several advantages over other explanations of the theory of mind: It can easily explain some behaviors in children at much earlier ages than other theories, and it is a much more economical explanation. In addition, it has a high biological evidence, with mirror neurons being a good candidate supporting the validity of this theory.

**Mirror neurons** are a class of neurons, discovered in rhesus monkeys, that fire both when the monkey performs a motor action, such as grasping an object, and when it observes another individual (monkey or human) performing the same or a similar action. However, they do not fire when the monkey only observes the object or the hand mimicking the grasping without a target object (Gallese et al., 1996). Neurophysiological and brain imaging experiments have shown strong evidence that a circuit analogous to the mirror neuron system from monkeys exists in humans (Rizzolatti and Craighero, 2004). This way, since mirror neurons fire both when we observe and when we perform an action, they are believed to be involved in our understanding of the states and actions of others, by mirroring the observed actions in our brains as if they were being performed by us (Gallese et al., 2004). This same mechanism seems to be also involved in other functions, such as imitation, as it provides motor copies of others' actions (Iacoboni et al., 1999). There is also evidence that mirror neurons take part in intention understanding: Research has shown that observing an action in a context that allows us to understand the intention of the action activates mirror neuron areas that observing the action without the context does not (Iacoboni et al., 2005). This way, several authors have suggested that mirror neurons are the basis for the theory of mind, supporting the simulation theory (Gallese and Goldman, 1998).

## 3. The Problem of Activity, Plan, and Goal Recognition

### 3.1. Problem Definition

Having had this overview about the mechanisms that are believed to describe action, plan and intention recognition in humans, we are ready to define the corresponding problem in machines, as well as to introduce its main challenges. We begin defining the concepts of activity, plan and goal recognition in the context of artificial intelligence (Sukthankar et al., 2014; Keren et al., 2019):

- **Activity recognition** refers to the problem of analyzing and adequately labeling low-level data from humans or other autonomous agents performing some action (Vrigkas et al., 2015; Jobanputra et al., 2019). This task usually involves processing noisy low-level sensory input streams, looking for patterns of interest in these data, discretizing them into meaningful subsequences and labeling each of these temporal subsequences. Sometimes it is also referred to as *behavior recognition*. The decrease in the sensor costs, together with the advancements in machine learning and big data and the spread of wearable devices, have boosted this field in the recent years and brought it to the forefront of research in computer vision and ubiquitous computing. Typically, these algorithms work with data coming from video cameras, accelerometers, motion capture sensors, RFID sensors, smart badges, Wi-Fi or Bluetooth signals, or GPS sensors, among others. These data are used to recognize very different types of activities, from daily-life activities, such as walking or sitting to more application-specific activities, such as those performed by a factory worker or a football player. A typical task within the problem of activity recognition is **action recognition** (or *action understanding*). Action recognition deals with recognizing short spatiotemporally localized actions or events (Poppe, 2010). This way, action recognition tries to segment the most elementary or primitive component of the activity (e.g., picking a dish) while activity recognition may work with longer sequences (e.g., washing dishes).
- **Goal recognition** refers to the problem of inferring the intention of humans or other autonomous agents by observing a set of actions performed by those agents or their effect on the environment (Han and Pereira, 2013). This task usually gets as input an ordered sequence of action labels (which may come from an action recognition module) and possibly a set of conceivable goals, and outputs the goal label that best explains the observed actions (or a set of labels with their corresponding probabilities). This task is often also referred to as *intention recognition* or *intent recognition*. However, several authors discourage the use of these terms due to the ambiguity of the words *intention* and *intent*, which are defined in very different ways across fields, and even within the same field (e.g., Xu et al., 2009 included the whole plan as part of what they called *intention*). On the other hand, the term *intent recognition* (or *intent classification*) is especially used to refer to the particular task of understanding the intention of a person from a sentence in natural language, making it not the most appropriate to refer to the more general task of goal recognition. In addition to the apparent differences, goal recognition also differs from activity recognition in the predictive component: Goal recognition tries to infer the final objective of the agent, which will imply a set of future actions, while activity recognition focuses on the action occurring at that instant (Kelley et al., 2010).
- **Plan recognition** is the problem of understanding the goal of humans or other autonomous agents, as well as the set of actions that have been or will be performed by those agents to reach that goal, given a set of observed actions performed by those agents (Carberry, 2001). This task has several things in common with the goal recognition task, but it is more general: It includes the goal recognition task and complements it with the task of defining a structure with the set of observed and predicted actions that will lead to that goal, as well as their relationships. While it provides a more complete solution to the problem than goal recognition, goal recognition may be more appropriate for applications where we need a fast detection of the goal and the detailed plan is not relevant.

**Figure 1:**
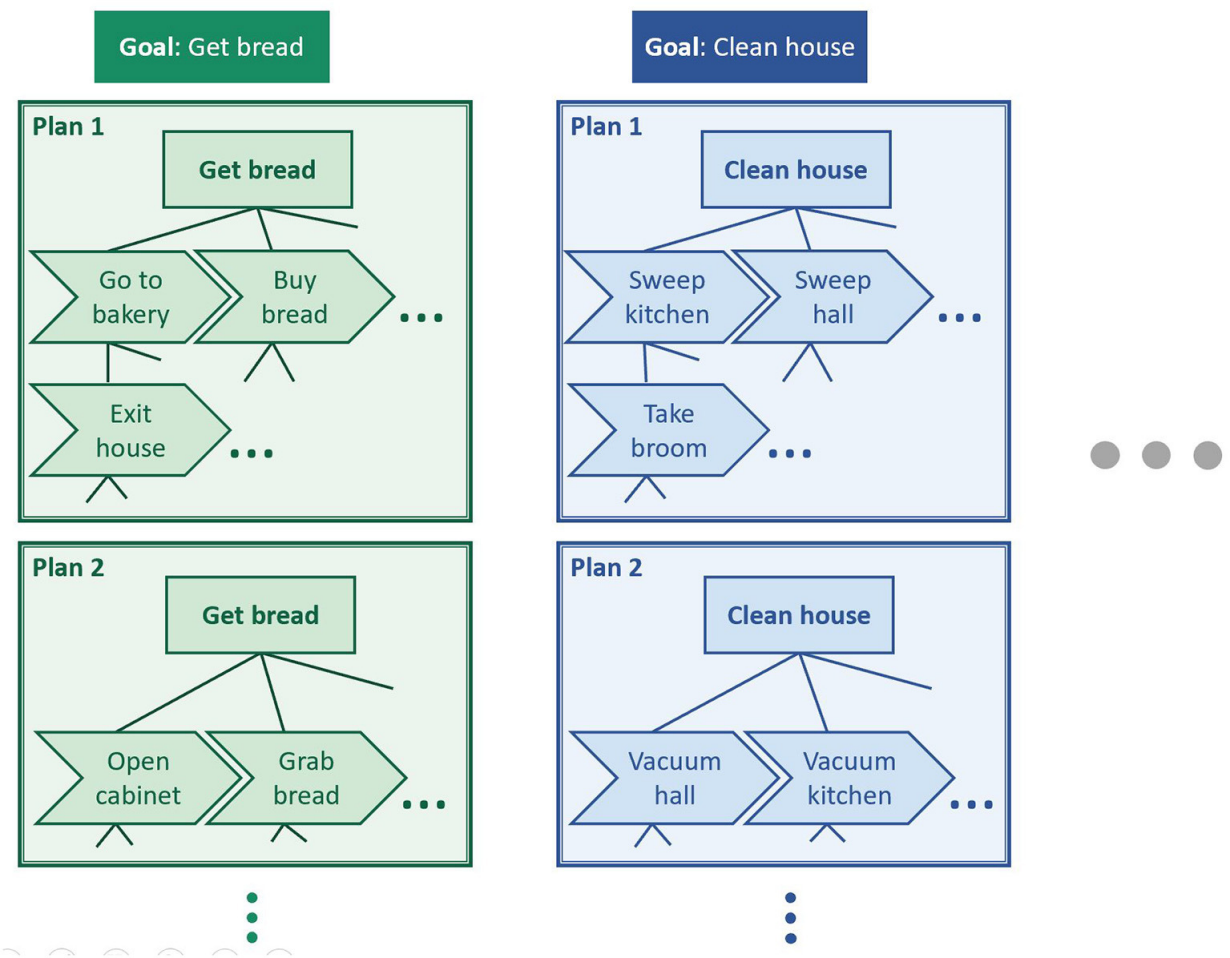
Illustration of the concepts of action, plan, and goal. A same goal may be achieved through different plans. These plans are often hierarchical structures whose elements can be seen as actions from the point of view of the higher-level elements, and as goals from the point of view of the lower-level elements.

It should be pointed out that, however, in many cases, recognition problems do not clearly belong to one of the classes described above, but they may have components of several of them. For example, in hierarchical architectures, the task of a layer may be seen as goal recognition with respect to the actions coming from the lower layer, but it may be considered action recognition from the point of view of a higher layer that takes its outputs as elementary actions. Figure 1 illustrates this idea of hierarchy. For example, task “Go to bakery” can be seen as an action that can be taken to achieve goal “Get bread,” but it can also be seen as a goal that can be achieved through a sequence of actions (“Exit house,” etc.). Similarly, “Exit house” can also be seen as a goal for lower-level actions (e.g., “Open door”), and “Get bread” as an action to perform higher-level goals (e.g., “Have lunch”). Figure 1 also shows how different plans formed of different sets of actions may lead to a same goal.

This way, we can define the general problem of activity, plan and goal recognition as the problem of, given a set of observations from the environment and/or the observed agent(s) (e.g., sensory streams, action labels, etc.), and, possibly, given a set of actions that can be performed (e.g., moving to get a better angle of view), finding the activity, plan, and/or goal(s) that best explain those observations. The problem is composed of three elements: the environment, the observed agent(s) [the actor(s)], and the observing agent (the observer) (Keren et al., 2019).

Regarding the input to the recognition systems, in addition to action labels or sensory streams, these systems can also get other types of input that, while less straightforward, may be decisive in the recognition task if used appropriately. For instance, information about the environment where the action occurs (e.g., kitchen, airport.) or about who is performing the action (e.g., a kid, a fireman.) can help recognition systems better evaluate what action, plan, or goal explains best the observations.

### 3.2. System Classification

So far, we have seen a possible way of classifying these recognition systems attending to their objective (i.e., activity, plan, or goal recognition). In fact, many other criteria can be adopted to classify these systems. For example, they are often classified in terms of the type of approach they take to address the problem. According to this, we can divide them into **logic-based** approaches, **classical machine learning** approaches, **deep learning** approaches, and **brain-inspired** approaches. We discuss these approaches in greater detail in section 4. Another characteristic that defines the type of recognition problem is the behavior of the observed agent toward the observer. In these terms, we can define three types of systems: **agnostic**, **adversarial** or **intended** (Carberry, 2001). In agnostic systems, the actor performs independently of the observer (he may even be unaware that he is being observed) (Shrager and Finin, 1982). In adversarial systems, the actor tries to deceive the observer, either by occluding the actions or by performing actions with the purpose of generating confusion (Avrahami-Zilberbrand and Kaminka, 2014). In intended systems, the actor tries to help the observer by, e.g., giving cues about the action being performed (Perrault and Allen, 1980). Similarly, recognition systems can be classified attending to whether the observing agent takes action and influences the actor or the environment with the purpose of making the recognition simpler. In this sense, recognition systems can be classified as **no intervention**, **offline intervention**, **online intervention**, or **direct communication** systems (Keren et al., 2019). In no intervention systems, the observing agent does not act in any way over the actor or the environment to make the recognition task easier (Avrahami-Zilberbrand and Kaminka, 2014). When these systems are also agnostic, they are known as **keyhole** systems. In offline intervention systems, the observer may introduce changes in the environment before the recognition process starts, with the purpose of making the recognition easier (Keren et al., 2014). In online intervention systems, the observer takes action over the actor or the environment during the recognition process, with the purpose of revealing some new information or causing a reaction over the actor that helps in the recognition task (Shvo and McIlraith, 2020). A common way to do this is through what is known as active perception (Bajcsy et al., 2018), which consists of taking action to increase or improve the input information (e.g., a robot moving to better see the ongoing action). In direct communication systems, the observer directly asks questions to the actor about the ongoing plans or goals, and reasons according to the answers (Mirsky et al., 2018).

**Figure 2:**
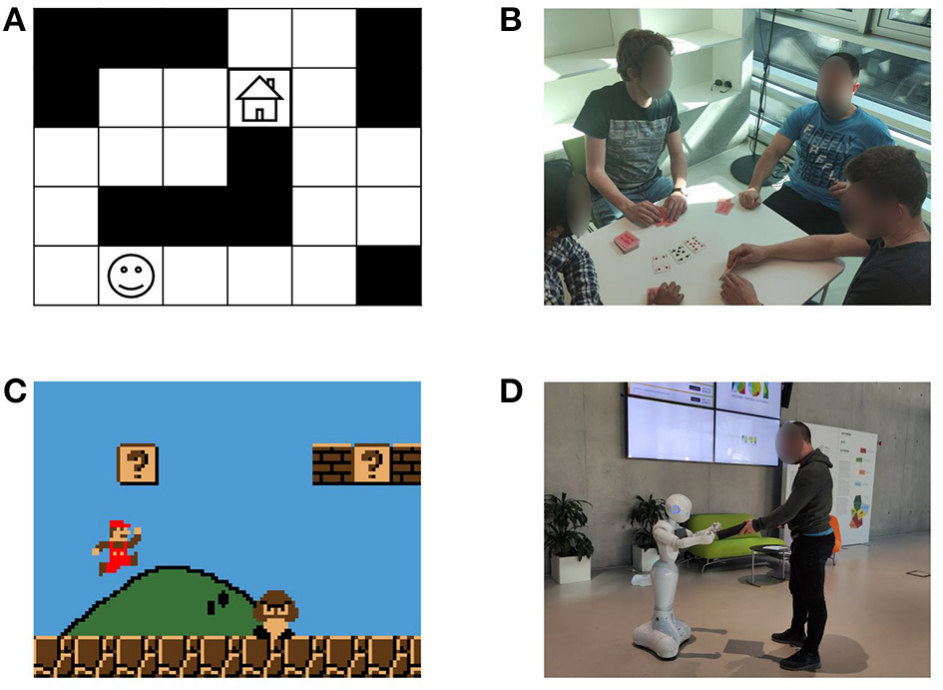
Examples of scenarios of different characteristics, **(A)** is a grid navigation scenario (agnostic, no intervention, fully observable, deterministic, discrete, single agent), **(B)** is a poker game (adversarial, direct communication, partially observable, stochastic, discrete, multiagent), **(C)** is a platform video game (agnostic, no intervention, partially observable, deterministic, continuous, single agent), **(D)** is a human-robot collaboration scenario (intended, online intervention, partially observable, stochastic, continuous, single agent).

Another way of classifying the recognition systems is attending to the characteristics of the environment. This way, the environment can be **fully observable** (when the observer perfectly knows its state) or **partially observable** (when the observer only gets a possibly noisy fraction of the information about the state of the environment) (Keren et al., 2016). It can be **deterministic** (if given a state and the action(s) performed over that state the next state is determined) or **stochastic** (if given a state and the action(s) performed over that state the environment may evolve to different states with different probabilities) (Wayllace et al., 2016). And it can be **discrete** or **continuous** (Kaminka et al., 2018). The systems can also be classified depending on whether the recognition process is done **offline** (the recognition is done at the end, with all the observations available) or **online** (observations are received incrementally, and the recognition is attempted to be done as soon as possible) (Vered et al., 2016). Another characteristic of these systems is whether the set of all **possible activities/plans/goals** is **known** or **unknown** by the observer (Zhuo, 2014). If it is unknown, the observer needs to be able to handle new classes of observations appropriately, e.g., by recognizing them as unknown activities and learning them, so that it can recognize them in the future. Finally, while most work in this area is directed toward recognizing the activity of a **single agent**, much research has been also done in **multiagent** systems (Saria and Mahadevan, 2004). This second type of recognition is typically used in cooperative applications, where the agent belongs to a team and needs to understand the role of the other members to perform best (Genter et al., 2011). It is also common when trying to understand the strategy used by the opponent in the military or sport domains (Laviers et al., 2009). Figure 2 shows some example scenarios classified according to the criteria just presented.

A completely different way of classifying these systems is according to the **applications** for which they were designed. Indeed, while many of the algorithms developed for activity, plan and goal recognition can be considered general-purpose, different applications often involve recognition problems with very different characteristics and types of input data (e.g., video, natural language, computer commands.), making algorithms more or less appropriate depending on the application. In addition, the “general-purpose” algorithms also require to be adapted to the particular problem, leading to more application-specific systems. There is a wide variety of applications where activity, plan, and goal recognition algorithms have been applied and proven to be useful. Some examples are smart homes (Skocir et al., 2016), personal agent assistants (Oh et al., 2014), human-robot interaction (Kelley et al., 2010), video surveillance (Poppe, 2010), video games (Ha et al., 2014), natural language understanding (Meng and Huang, 2018), assistive care for the elderly (Bouchard et al., 2007), software help systems (Horvitz et al., 1998), computer network security (Rahmat et al., 2018), decision support systems (Sengupta et al., 2017), and orthotics (Rebelo et al., 2013), among others. For example, assistive systems for the elderly need to understand the intention of the assisted person in order to anticipate and be able to help. Computer network security systems, on the other hand, need to analyze network activity and detect suspicious actions or actions performed with a malicious intention, and act accordingly.

### 3.3. Challenges

Having introduced some of the main characteristics that describe activity, plan, and goal recognition systems, we can now present a number of challenges that these systems need to overcome in order to complete their task effectively. Below we provide a non-exhaustive list of some of these challenges, as well as considerations that these systems need to take into account:

- Managing multiple competing hypothesis and uncertainty (Sadri, 2011)
- Dealing with previously unseen activities/plans/goals (Carberry, 2001)
- Variability among different instances of the same action/plan (Vrigkas et al., 2015)
- Predictive capabilities, or completing the recognition task before the action/plan is completed (Kelley et al., 2010)
- Incomplete knowledge and partial observability of the actor and the environment, as well as noisy input (Sadri, 2011)
- Interleaved plans or plans executed in parallel, either to achieve different goals or the same goal in alternative ways (Sadri, 2011)
- Interrupted plans (Armentano and Amandi, 2007)
- Actions belonging to more than one plan or contributing to more than one goal (Kautz and Allen, 1986)
- Actions performed by different agents to reach a common goal (Sadri, 2011)
- Non-rational actions, reactive (not goal-directed) actions, exploration actions, irrelevant actions, actions executed by error, or actions executed with the purpose of misleading the observer (Carberry, 2001; Sadri, 2011)
- Considering the temporal ordering of events (Kautz and Allen, 1986)
- Adapting to the agent being observed (Zhuo, 2017)
- Considering the context (Heinze, 2004)
- Expressivity and interpretability of the system output (Armentano and Amandi, 2007)
- Scalability to a greater amount of activity/plan/goal classes and to multi-agent systems, and adaptability to different environments (Carberry, 2001)

### 3.4. Other Existing Reviews

Before deepening further in the different types of approaches taken to tackle these problems, we can go through a brief overview of other literature reviews available on this topic. The *Introduction* chapter of the book *Plan, Activity, and Intent Recognition. Theory and Practice* (Sukthankar et al., 2014), for example, provides a short review of the history of the topic. However, since the book was published in 2014, it mainly focuses on logic-based and probabilistic approaches, but the more recent deep learning approaches are not considered. Vrigkas et al. (2015) address the topic of activity recognition focusing on applications that get as input still images or video sequences. Their work also goes through the low-level task and algorithms for extracting features of interest from images (which are out of the scope of our review). A more thorough overview on these kinds of activity recognition systems can be found on the book *Human Activity Recognition and Prediction* (Fu, 2016), which besides going through the different techniques, also introduces some general relevant concepts on activity recognition. Another review on the problem of activity recognition is that of Wang et al. (2019), in this case focusing on sensors different from cameras (e.g., body-worn sensors, etc.), and on deep learning approaches. Other reviews on activity recognition are Poppe (2010), Lara and Labrador (2013), and Jobanputra et al. (2019). On the other hand, Carberry (2001) describes in her review the problem of plan recognition, together with its main challenges and approaches to address it. While this study was published in 2001, it is a quite complete work, and many of the concepts and challenges commented are still relevant nowadays. Armentano and Amandi (2007) also provide a review on the topic of plan recognition, which, while focused on interface agents, can also be of interest to other application fields. Finally, Sadri (2011) reviews the main logic-based techniques used to approach the problem of goal recognition.

These reviews, in general, either focus on the problem of plan and goal recognition, going through the main existing logic-based and probabilistic solutions, or focus on the problem of activity recognition, covering machine learning approaches (and, in recent years, specially deep learning approaches). This goes in line with what can be found in the research studies themselves, with a clear separation between those subfields. However, to the best of our knowledge, there are no reviews that cover the whole problem of activity, plan, and goal recognition together with the most common approaches in a comprehensive way. Indeed, none of the consulted reviews includes logic-based, classical machine learning and deep learning solutions altogether. Furthermore, we have found no review covering the most common brain-inspired methods to approach this recognition problem, which is a relevant topic when it comes to human-robot interaction. In this review, we try to fill this gap, by describing the whole problem of activity, plan, and goal recognition, presenting the main challenges and characteristics with which it can be described, and going through the different types of approaches used to address it. This view of the problem as a whole may also inspire researchers to think of new approaches that address the complete problem in a unified way, instead of focusing on just a part of it.

## 4. Approaches to the Problem of Activity, Plan, and Goal Recognition

This section describes in more detail the different types of approaches that have been taken to address the problem of activity, plan and goal recognition. As expressed in section 3.2, we can classify these algorithms according to the type of approach into four different classes: logic-based, classical machine learning, deep learning, and brain-inspired.

### 4.1. Logic-Based Approaches

Approaches based on logical reasoning (Stuart and Norvig, 2016) have mainly focused on the problem of plan and goal recognition, even though there have been also attempts to tackle the activity recognition problem. In fact, the first attempts that were made to address the problem of activity, plan and goal recognition took this type of approach (Schank and Abelson, 1977; Schmidt et al., 1978). Following the predominant tendency in artificial intelligence at these early times, researchers defined a set of domain-dependent rules that tried to capture the relevant knowledge that allowed the system to infer conclusions through deduction (Sukthankar et al., 2014). As occurred in other areas, this approach soon showed to be very limited in several ways, particularly in maintainability and scalability. Some years later, a representation of plans as tree graphs was proposed, where the plan was represented as the top-level node of the tree, and the actions in which it was decomposed, as the child nodes (Kautz and Allen, 1986). This work gave some structure and coherence to the field, and the conceptual framework proposed is still relevant today (Sukthankar et al., 2014). However, these early techniques still suffered from their purely deductive inference method: If there were more than one possible plan or goal compatible with all the observations, the system was unable to decide which one was the most likely explanation.

Since then, mainly two kinds of **reasoning** have been employed to cope with this issue of hypothesis selection: abductive reasoning and hybrid logic-probabilistic reasoning. **Abductive reasoning**, or abduction, is a form of logical inference that tries to find the simplest or most likely conclusion that can explain some given observation(s) (Josephson and Josephson, 1994). This way, unlike deduction, the conclusions reached through abduction are not positively verified, but are rather plausible conclusions understood as best explanations. This type of reasoning allows the inference system to choose among several hypotheses to explain an observation. In fact, hybrid logic-probabilistic approaches can be considered a special case of this kind of abduction. Sohrabi et al. (2016), for example, associated a cost to each of the possible plans, as well as to the noisy or missing observations. By summing these values for a given observation and candidate plan, they obtained the weight for that hypothesis. The plan with the lowest weight was the most likely one. Jarvis et al. (2004), on the other hand, developed a terrorist activity detection system based on the definition of two concepts, called “frequencies” and “accuracies,” that were associated to each of the actions. An action with a lower “frequency” or higher “accuracy” associated would be considered more relevant when it was observed.

As we just mentioned, **hybrid logic-probabilistic reasoning** can in fact be considered a special case of abductive reasoning which explicitly deals with probabilities. However, we consider it separately from pure abductive reasoning because, even though it is conceptually similar, it combines logic inference methods with probabilistic inference methods. These approaches have the advantage that they keep the expressivity of logic solutions while being able to handle uncertainty in a probabilistic way. There are different ways of combining those two types of reasoning. Some examples of commonly used hybrid models are relational Markov models and Markov logic networks (Kautz, 2011). Relational Markov models (RMM) generalize Markov models by allowing states to be of different types and to be hierarchically structured, while Markov logic networks (MLN) combine ideas of Markov networks with first-order logic, enabling probabilistic inference. Pereira and Han (2009) proposed an intention recognition system for elder care based on causal Bayesian networks. These networks established probabilistic relationships among causes, intentions, actions and effects, and were used to extract the most probable intentions. The plausibility of these intentions in the given situation was then checked through a logic plan generator. Raghavan et al. (2014) proposed a very different approach, which lied on the framework of statistical relational learning (SRL). The formalisms within this framework typically describe relational properties using first-order logic, while they handle the uncertainty through probabilistic models. In the paper, they proposed an extension of Markov logic networks and Bayesian logic programs to adapt them to abductive reasoning and perform plan recognition.

Some authors combine these abductive or hybrid reasoning techniques with some kind of logic-based **causal theory**, such as situation calculus or event calculus (Stuart and Norvig, 2016). These formalisms allow reasoning in dynamic domains through the introduction of temporal constraints, which enable the definition of temporal properties, such as preconditions or effects. Quaresma and Lopes (1995) combined abductive reasoning with event calculus and some concepts from the theory of mind (commented in section 2) to address the problem of recognizing plans and intentions behind speech acts. Their model described the mental state of the observed agent in terms of intentions and beliefs, and reasoned according to that mental state and to the effects the agent was believed to expect from the actions.

So far, we have focused on the type of reasoning to classify the logic-based approaches to the problem of activity, plan and goal recognition. These approaches can also be classified in terms of **how knowledge is represented**. Attending to this, we can divide them into two types: plan-library based and domain-theory based (Stuart and Norvig, 2016; Vered and Kaminka, 2017). Approaches based on **plan libraries** are sometimes referred to as *plan recognition as parsing*, because plans are usually represented as a hierarchy of lower-level actions, and the problem is reduced to finding the best fit of the observed actions into those plans. There are several ways of representing the knowledge in plan libraries. A common one is using hierarchical task networks (HTN) (Stuart and Norvig, 2016). Hierarchical task networks represent tasks as a set of subtasks and constraints on them or among them. This way, tasks can be iteratively expanded until primitive tasks are reached, which would correspond to observable actions. Myers (1997) used hierarchical task networks to recognize the high-level goal of a user of a collaborative planning system by observing a partial plan (a partial set of actions). Once a complete plan to which that partial plan belonged was identified, the system could complete the remaining actions. Another usual way of representing the knowledge in plan libraries is using grammars (Stuart and Norvig, 2016). Grammars define a set of symbols and production rules that describe how they can be combined, enabling the generation of hierarchical tree-like structures. They were originally used in the parsing of natural language, but they have also shown to be useful in plan recognition (note the similarity between both tasks, dealing with hierarchical and sequential data). When the grammar rules are described through probabilities, they are known as stochastic grammars. Geib and Goldman (2009) proposed a probabilistic plan recognition system based on plan tree grammars that was able to handle interleaved plans, partially ordered plans, and partial observations.

Regarding **domain-theory** based approaches, they are often known as *plan recognition as planning*. In these approaches, off-the-shelf planning systems are used to generate candidate plans for the observed agent. These planning systems generally rely on planning languages, such as STRIPS or PDDL (Stuart and Norvig, 2016), which allow them to describe the state of the environment and the effects of the possible actions, as well as to reason over that knowledge in order to develop candidate plans that lead to the achievement of given goals. These candidate plans are then weighted by the plan recognition system according to the observations obtained, and the most likely plan and/or goal is selected according to those weights. When the planning system used relies on Bayesian inference to reason, these recognition approaches are sometimes referred to as Bayesian inverse planning systems. Ramirez and Geffner (2009) were the first ones suggesting the plan recognition as planning approach. They proposed an approximate planning method that generated an acceptable set of plans to the possible goals, and that was able to scale well. Pereira et al. (2020) went through some of the main state-of-the-art domain-theory based techniques to goal recognition focusing on the concept of landmarks, which are states or actions through which the observed agent needs to go in order to achieve a certain goal. They proposed a landmark-based goal recognition approach that was able to perform faster than other systems, while having a comparable accuracy.

#### 4.1.1. Summary

As a summary, we can say that logic-based and hybrid logic-probabilistic approaches are probably the most commonly used ones in the literature to deal with the problem of plan and goal recognition. Logic systems present several advantages that are very useful for this problem. One of the main ones is that they are very good at working with highly structured representations. Logic representations can define different kinds of relationships among entities, such as preconditions, mutual exclusions, or decompositions, which are very useful for the problem at hand. Besides, these logic relationships allow these systems to generate new plausible candidate plans online, which can be compared to the actual observations. A second advantage is that logic representations are highly expressive. This way, these systems allow their designers and users to easily understand the meaning of their output, as well as how they have reached that conclusion. In addition, when combined with probabilistic reasoning techniques, logic-based systems can reduce some of their main weaknesses, such as not being able to handle uncertainty.

However, even when combined with probabilistic reasoning methods, they still present some disadvantages. For example, logic systems are very rigid: They define entities in terms of a set of properties and relations, and observations need to fit them perfectly in order to be considered such entity. In general, however, real-world “entities” are more ambiguous and require more flexible approaches. Even when combined with probabilistic methods, the logic-based component of the system usually keeps this rigidity. This is particularly relevant in more uncertain environments, such as those partially observable, where the knowledge of the observed agent is unknown, or where interleaved or interrupted plans can occur. Another disadvantage of these systems is that they usually require the designer to introduce manually the domain knowledge upon which the system will reason. This limits the use of these approaches to applications where the necessary knowledge can be expressed in such way. Indeed, even if the system is able to generate new candidate plans online, they will always be based on that limited domain knowledge, and plans that fall out of the scope of this domain will not be recognizable. In addition, in domains that are complex enough, introducing all the knowledge manually will require much domain-expert effort and will be prone to errors. This makes these systems bad at scaling and generalizing. Finally, these systems usually assume that the observed agent is rational, and try to find the optimal plan that best fits the observations. However, humans often act in non-rational ways (Dreyfus, 2007), making these systems not so appropriate.

### 4.2. Classical Machine Learning Approaches

In the previous section, we saw some examples of hybrid logic-probabilistic approaches to the problem of activity, plan and goal recognition. In fact, probably most of the proposed classical machine learning solutions to this problem are based on probabilistic systems, such as Bayesian networks or Markov decision processes. Therefore, in this section we will focus mainly on these probabilistic approaches, depicting at the end also some non-probabilistic techniques that have been used for this problem. As we just said, many of these approaches are based on different types of **Bayesian networks** (BN) (Stuart and Norvig, 2016), such as dynamic Bayesian networks or hidden Markov models. Bayesian networks are generative probabilistic graphical models that represent random variables as nodes and conditional dependencies as arrows between them. They can provide the probability distribution of any set of random variables given another set of observed variables. In the case of activity, plan and goal recognition systems, those random variables can represent, e.g., low-level observations, actions at different hierarchical levels, or final goals. Due to the temporal nature of the problem, the Bayesian networks used typically take the form of **dynamic Bayesian networks** (DBN) (Stuart and Norvig, 2016). Dynamic Bayesian networks are a kind of Bayesian network that represents its variables at different time steps, as well as the conditional dependencies across time steps. For example, Liao et al. (2004) built a dynamic Bayesian network with a hierarchical structure that could infer the transportation modes or destination goals of a user from low-level GPS sensor measurements, as well as recognize abnormal or unknown activity. They used particle filtering to infer across the network.

A particular well-known type of dynamic Bayesian network that has been commonly used for the problem of activity, plan and goal recognition is **hidden Markov models** (HMM) (Stuart and Norvig, 2016). Hidden Markov models assume that an observable variable exists which depends on a hidden variable (i.e., the state), which depends on itself at the previous timestep. This allows to, given a sequence of observations, find the most likely hidden state(s). The use of this type of Bayesian network is quite spread in problems dealing with sequential data, such as natural language processing, and well-known techniques exist to infer on it. Kelley et al. (2010) used hidden Markov models to model actions and goals within a human-robot interaction scenario. They built one hidden Markov model for each possible goal, with the hidden states representing the possible actions. However, hidden Markov models are quite simple models, with little structure, while as we argued earlier, activities, plans, and goals often have a relatively complex and hierarchical structure. Some extensions of hidden Markov models exist that where designed with hierarchical structure in mind and that have been successfully applied to the problem of activity, plan and goal recognition, such as layered hidden Markov models (LHMM) Oliver et al. (2002) or hierarchical hidden Markov models (HHMM) (Bui et al., 2004). Bui et al. (2004), for example, proposed an extension of the hierarchical hidden Markov model that allowed two states to share the same child (e.g., the same action belonging to two different plans), and applied it to the problem of action and plan recognition in an airport scenario.

Another popular probabilistic model used in sequential classification problems, and that has been used mainly for activity recognition, is linear chain **conditional random fields** (linear chain CRF) (Stuart and Norvig, 2016). Linear chain conditional random fields are discriminative probabilistic graphs that are applied and can deal with similar problems as hidden Markov models. However, they are more powerful, since they can model everything that hidden Markov models can and more. Zhao et al. (2010) proposed an activity recognition system that extracted a set of features from patterns found in inertial data to feed a linear chain conditional random field. Similar to hidden Markov models, linear chain conditional random fields are also quite simple in terms of structure. Liao et al. (2007) used a two-level so-called hierarchical conditional random field (hierarchical CRF) to predict a person's activity (first level) and the place at which the activity was taking place (second level) based on GPS data.

A different probabilistic approach to those already mentioned is modeling the observed agent as a **Markov decision process** (MDP) (Stuart and Norvig, 2016). Markov decision processes model an agent decision process in a system where the transition between states, as well as the rewards obtained by the agent, depend probabilistically on the actions taken. This type of approach is sometimes referred to as inverse reinforcement learning. Oh et al. (2014) proposed a proactive assistant agent for domains such as emergency response and military peacekeeping operations, which modeled the observed agent as a Markov decision process, allowing the system to infer the agent's goals, predict the following actions and provide assistance accordingly. Markov decision processes assume that the state of the system is known to the agent. However, this is not the case in general. Partially observable Markov decision processes (POMDP) (Stuart and Norvig, 2016) deal with this by maintaining a probability distribution over the set of possible states. Baker and Tenenbaum (2014) proposed a probabilistic theory of mind model where the mental state of the observed agent (beliefs and desires) where modeled as probabilistic distributions within a partially observable Markov decision process. Using Bayesian inference, the system could estimate the belief state and reward function of the agent.

As we said earlier, other **non-probabilistic** classical machine learning techniques have also been used, mainly for the problem of activity recognition, such as support vector machines (SVM), decision trees, k-nearest neighbors (KNN), or shallow artificial neural networks (shallow ANN) (Lara and Labrador, 2013; Stuart and Norvig, 2016). Support vector machines are binary classifiers that divide the feature space through a hyperplane, with each of the resulting subspaces corresponding to each category. While they are intrinsically linear, they can be extended to perform non-linear classification, as well as to work with more than two classes. Samanta and Chanda (2014) used support vector machines to classify human activities represented through space-time features extracted from video data, and tested their approach on several standard datasets. Decision trees are classification algorithms that use a tree-like model of decision. Each node is characterized by some criterion according to which samples are sent to one of its subnodes, until samples reach an end node or leaf, which has a class assigned. Fan et al. (2013) used decision trees to classify daily-life activity data coming from the accelerometers of a smartphone. The system was able to classify the data successfully independently of the actual location of the smartphone.

Finally, few **unsupervised** learning systems have also been proposed to address the problem of activity recognition. These systems, however, while unsupervised, are quite limited in performance and in the types of applications for which they can be useful. For example, Vahdatpour et al. (2009) proposed an unsupervised system for motif (recurring pattern) learning and detection for activity recognition using clustering techniques. Polyvyanyy et al. (2020), on the other hand, proposed an unsupervised probabilistic goal recognition system based on process mining techniques.

#### 4.2.1. Summary

In summary, classical machine learning approaches (and, in particular, probabilistic approaches) to the problem of activity, plan and goal recognition have shown as their main advantage being good at handling uncertainty. This makes them useful to deal with situations that are common in real environments, such as handling interrupted or interleaved plans, coping with partial observability or noisy data, or even dealing with non-rational agents or dynamic domains. These advantages are especially noticeable in the task of activity recognition, in which logic-based approaches are often not appropriate. In addition, classical machine learning approaches do not require a full manual introduction of the domain knowledge, as they can learn the parameters (e.g., probabilities) given enough training data. On the other hand, well-studied hierarchical networks exist that provide structure to the models, minimizing one of the limitations of these approaches.

However, classical machine learning approaches have also several disadvantages. First, these methods are less expressive than logic-based ones, and it is often hard to understand how one of these models has reached a certain conclusion. In addition, while it is true that some classical machine learning systems scale well (see section 4.3), probabilistic systems in general do not: As these systems become more complex, more variables and dependencies among them need to be modeled, estimated, stored and properly used. Besides the fast growth in the number of dependencies, the structure of the network also needs to be designed. While this structure can also be learned from data, taking this approach requires a higher amount of data, and leads to difficult-to-understand networks and relationships between variables. On the other hand, even though well-known hierarchical structures exist, they are still very limited on the types of relationships among variables they can model, and their structure is also very rigid. Regarding non-probabilistic systems, not dealing explicitly with probabilities also comes with some limitations, as they do not provide information on the certainty of the obtained output. This can be an issue in applications where having a “best guess” is not enough, but information on the confidence of that guess is also required. Online recognition systems may also need such information to understand if the ongoing action/plan/goal has already been recognized. Finally, classical machine learning approaches in general, similar to logic-based ones, are not designed to learn online new activities, plans or goals, something that may be necessary in real environments.

### 4.3. Deep Learning Approaches

The quick introduction and success in recent years of deep learning approaches into the fields where more classical machine learning techniques were commonly used has also affected the field of activity, plan and goal recognition. In particular, deep learning (Goodfellow et al., 2016) is becoming one of the main technologies to deal with the problem of activity recognition (Wang et al., 2019), and it has also been used for plan and goal recognition. Among the different deep learning approaches, the most commonly used one is **deep neural networks** (DNN) (Goodfellow et al., 2016). Deep neural networks usually consist of a set of layers composed of several neurons. The input data goes from the input layer, through all the hidden layers until it reaches the output, and it is processed in each layer according to a function that has been learned. A deep neural network architecture that is simple in terms of design and that has been used for activity recognition is the **deep feedforward fully-connected neural network**, where there are no cyclic connections between the neurons and all neurons of a layer are connected to all neurons of the next layer. Hammerla et al. (2016) designed a five-hidden-layer network to recognize activities from data coming from wearable sensors and compared this architecture to other popular deep architectures, such as convolutional neural networks and long short-term memory networks. These last architectures, in general, outperformed the first one, and were able to converge to a system with an acceptable performance much faster.

**Convolutional neural networks** (CNN) (Goodfellow et al., 2016) are probably the most commonly used deep approach to the problem of activity recognition. These networks are very popular when processing images or temporal data because they extract local patterns from elements close in the image or in time. When applied to temporal data, the input data is usually divided into time windows, and these windows are classified by the network. In the case of activity recognition, these networks have shown to be very effective when working with prolonged and repetitive activities, such as walking or running (Hammerla et al., 2016). Bevilacqua et al. (2018) used and compared different configurations of inertial sensor data to train different convolutional neural network architectures to classify physical activities. Ronao and Cho (2016) tried convolutional neural networks of different depths and kernel sizes to classify activities coming from smartphone sensors.

Another deep architecture that has been used for the problems of activity, as well as plan and goal recognition is recurrent neural networks (RNN) (Goodfellow et al., 2016). In these architectures, cyclic connections between the neurons exist, allowing the network to have memory and to be aware of the context of the data. This makes them very useful to process sequential data. Among the different recurrent architectures, the most widespread one is **long short-term memory networks** (LSTM) (Goodfellow et al., 2016). These networks are designed so that they can decide when to update their memory depending on the context and they can learn long-range relationships in the input data. This is an advantage over convolutional neural networks, which can only count on the information in the temporal window for the classification. In the case of activity recognition, recurrent neural networks have demonstrated to be very useful at classifying activities that are short in duration but have a natural ordering, thanks to their ability to take the context into account (Hammerla et al., 2016). Amado et al. (2018a) proposed the use of long short-term memory networks for a goal recognition task dealing with sensory input data, requiring much less manual introduction of domain knowledge than other state-of-the-art goal recognition approaches. Ordóñez and Roggen (2016) combined convolutional neural networks with long short-term memory networks for the task of activity recognition. They used the convolutional networks for low-level feature extraction at the first layers of the network, followed by long short-term memory networks that could capture the temporal dynamics and context of the observations.

Finally, there are deep architectures that can learn the low-level feature extraction functions in an unsupervised way, such as **autoencoders** or **deep belief networks** (DBN) (Goodfellow et al., 2016), requiring less labeled data to achieve acceptable performances. Zhang et al. (2015) implemented a deep belief network for activity recognition that could run and be trained in a smartphone, showing that these networks can be computationally very efficient. Min et al. (2014) used stacked denoising autoencoders to model the goals of players in an open-ended digital game, improving considerably the accuracy of other state-of-the-art models. On the other hand, few-shot learning and zero-shot learning techniques have recently gained interest for the task of activity recognition systems. Indeed, these techniques are gaining popularity in the last years, especially in the labeling of images and videos. **Few-shot learning** allows systems to learn to recognize new classes from very few labeled samples. During this process, it is also important that they do not forget the previously learnt classes. A common way to do this is by training a deep network to extract a set of representative features that are later used in a nearest neighbor-like classifier (e.g., a Siamese neural network) (Sheng and Huber, 2019). Data augmentation techniques, i.e., generating new synthetic data out of the existing ones, and overfitting avoidance techniques are also commonly used (Dwivedi et al., 2019). However, most of the work in few-shot activity recognition has focused on images, and only few studies exist working with videos (e.g., Xian et al., 2020). In addition, the performance achieved in these systems is far from satisfactory. **Zero shot learning**, on the other hand, allows systems to label, or associate semantic meaning, to classes that were not present in the training data. Note that these systems do not actually learn during this recognition process, they just describe those unknown classes in terms of what they already knew. Zero-shot activity recognition systems are usually formed of two components: a visual feature extractor that describes input images and videos in terms of features, and a semantic feature extractor that maps semantic side information (e.g., in the form of descriptive sentences) to features. The training data is used to learn a mapping function between these two feature spaces, allowing the system to describe the images and videos in terms of the side information. This mapping is expected to be able to generalize to unseen classes (Junior et al., 2019). However, the zero-shot activity recognition systems proposed up to date still show poor performance.

#### 4.3.1. Summary

To sum up, deep learning models have several strengths that have made them the de facto algorithms in different fields, and that also apply to activity, plan and goal recognition. One of the main advantages of these approaches is that they are able to learn very different patterns, and their hierarchical architecture in layers allows them to extract features from the input data at different levels of abstraction, which are then used by the next layers. This also makes the training faster and the data requirements smaller, as higher-level layers usually require similar lower-level features. This way, they are very flexible and hierarchical by nature, something very useful for the problem of activity, plan and goal recognition. In addition, the input data to the network is often the sensor data itself, and the first layers of the network learn automatically the best feature extraction functions from the sensor data for the task at hand during the training process. This is an important advantage for activity recognition, and also for plan and goal recognition when they deal with sensor data. Most logic-based and classical machine learning approaches use manually designed features from the sensor data as input for the algorithms. These features require human effort and domain knowledge, are not generalizable, and may not be optimal. Finally, similar to classical machine learning models, deep learning approaches are good at dealing with uncertainty and with partial and noisy information, as long as those characteristics also exist in the training data.

One of the main disadvantages of deep learning approaches is that they usually require large amounts of labeled training data to perform successfully. This is related to the fact that, to achieve their high flexibility, they have many parameters that need to be learnt. Even using unsupervised approaches as those commented above to learn low-level features, they still require large labeled datasets. Few-shot learning approaches, on the other hand, cannot be considered satisfactory in terms of performance. This is a relevant limitation in the problem of activity, plan and goal recognition, as the different available datasets are built using different types of sensors, in different configurations and labeled according to different activities or goals, and therefore it is hard to combine them and exploit them together. Another limitation of deep architectures is that they usually require high computational power, especially on the training phase. Besides, deep networks are difficult to interpret, and they are often seen as black boxes, with the reasons that lead them to a particular conclusion being unknown to their designers or users. This can be a limitation especially for plan recognition, where the description of plans usually requires high expressivity. In addition, most deep architectures are non-probabilistic, which brings a set of difficulties, as we discussed in section 4.2.1 (e.g., not providing information on the certainty of the outputs). Finally, the most common architectures are not designed to learn online new classes, and even when they can, they require labeled data. This is a limitation in real environments. In this sense, note that zero-shot learning systems, besides performing poorly, do not learn the newly observed activities. This way, they are not able to adapt and learn new useful features online, and neither to learn to predict next actions, something often required within the problem of activity, plan and goal recognition.

### 4.4. Brain-Inspired Approaches

The approaches seen so far use algorithms and techniques that are, in general, well-established within the artificial intelligence and machine learning communities. In fact, most of the work in this area has been done using these types of approaches. However, as we have just seen, these standard approaches present some relevant limitations for the problem at hand, especially when dealing with real environments, where being able to handle ambiguity, partial information and unknown actions or goals is very important. Therefore, several authors have tried alternative approaches to address this problem, inspired by the fact that the human brain is able to deal with these issues in a very effective way. These approaches have especially been used in human-robot interaction applications.

As we explained in section 2, mirror neurons are believed to be a key element in our ability to understand the actions and intentions of others. This way, several **models of mirror neurons** have been used for the tasks of action and goal recognition, as well as for imitation. For example, mirror neurons have been often modeled as auto-associative memories. These memories learn patterns of actions when executing movements, associating the actions to the corresponding observations during execution. Then, when the execution of a similar action is observed, the action is recognized (Kuniyoshi et al., 2003). A more elaborated model of mirror neurons is MNS2 (Mirror Neuron System 2) (Bonaiuto et al., 2005), which models several regions of the brain as recurrent neural networks, and is able to learn different actions through self-observation to later recognize them on others. A quite different model of mirror neurons is the Mental State Inference model (Oztop et al., 2005). This model is not based on the association of self-performed actions with their corresponding observations, but instead, given some observations, simulates the actions that would correspond to the possible intentions and compares them to the actual observations, choosing the intention with the best prediction as the most likely one.

There are also several models that, while they do not try to mimic directly the functioning of the mirror neurons, show several similarities, and have shown to be useful on the tasks of action and goal recognition, as well as imitation. One of these models is **MOSAIC** (Modular Selection and Identification for Control) (Haruno et al., 2001). MOSAIC is a learning control system that consists of several predictor-controller modules. The controller part of each module generates motor commands that are used by the predictor part to simulate the movements that those commands would imply, and those simulated movements are then compared with the observed ones. This way, modules with better predictions become more influential. Another popular system with a similar architecture of inverse-forward blocks is **HAMMER** (Hierarchical, Attentive, Multiple Models for Execution and Recognition) (Demiris and Khadhouri, 2005). The modules in this system can be combined to form more complex modules, achieving a hierarchy of modules that can operate at a higher level and can represent more abstract or full behaviors. A limitation of these architectures is the conflict between generalization and segmentation that arises when motor primitives overlap, where generalization leads to representing many similar primitives with the same module, while segmentation requires that different primitives are represented in different modules. **Multiple timescales recurrent neural networks** were designed as an attempt to overcome this issue (Yamashita and Tani, 2008). Instead of using separate modules, they work with self-organization mechanisms and neurons working at different timescales, which leads to the emergence of a functional hierarchy among actions. Finally, some action recognition systems directly implement some form of explicit visuo-spatial perspective-taking (i.e., they project the point of view of the observed agent onto their own). Alkurdi et al. (2018), for example, projected the frame of reference of the observed agent into that of the observer, to then feed an action recognition system that was built upon a cognitive framework known as dynamic field theory (Schöner and Spencer, 2016). Their system first detected the object of interest over which the action would be performed, to then compare the observed trajectory to those corresponding to the possible actions over that object.

Many other brain-inspired algorithms and **cognitive architectures** exist, such as Adaptive Control of Thought-Rational (ACT-R) (Ritter et al., 2019), Soar (Laird, 2012), Adaptive Resonance Theory (ART) (da Silva et al., 2019), or Hierarchical Temporal Memory (HTM) (Hawkins et al., 2016), that could be used to approach the problem of activity, plan and goal recognition. These architectures try to mimic in different ways and integrate the existing known mechanisms in our brain (e.g., different types of memory, learning, attention, etc.) to achieve cognition. For example, HTM models neocortex layers as a set of nodes that learn following a Hebbian-like rule, and that are activated according to a sparse coding paradigm. Hebbian learning is a simplified model of how neurons in the brain learn. It works in an online and unsupervised way, and has shown to be very practical at learning and extracting patterns. Sparse coding is a representation method that is believed to occur in the brain, in which just a small set of all the elements are active for each represented concept. These elements may represent the presence of certain features in the represented concept. This method has shown to be very robust and useful at representing new concepts in terms of previously learned features. As an example of a recognition system, Oltramari and Lebiere (2014) built an ACT-R-based system for the recognition of actions in a video surveillance application. However, most of these brain-inspired frameworks and architectures have been little explored for the problem at hand, and it is therefore difficult to predict how effective they and their mechanisms would be for this problem. An overview and comparison of some of the best-known existing cognitive architectures can be found in Kotseruba and Tsotsos (2020).

#### 4.4.1. Summary

We conclude that, due to the great variety and diversity of brain-inspired algorithms and architectures, it is difficult to outline general advantages or disadvantages of these methods over those described in the previous sections. However, we can comment on the mechanisms that are known to exist in the brain and that are often integrated in these frameworks. One of these mechanisms is Hebbian-like learning, which, as we said previously, is a form of unsupervised and online learning method that is good at finding patterns. This can be particularly useful for real open environments and, in general, to relax the human effort requirements for training. Related to this, sparse coding can also help on the learning of new concepts online and in few shots. This adaptability also endows these systems with tools to handle uncertainty and dynamic environments. In addition, these algorithms often work in a predictive way, which is a usual requirement for plan and goal recognition systems. Some brain-inspired algorithms also implement self-organization mechanisms, which, besides working in an unsupervised manner, can improve the performance and interpretability of the system. Similarly, attention mechanisms are often included in these frameworks, which can also improve the interpretability and performance of the system, besides reducing the computational requirements. Finally, mimicking particular structures of the brain, such as the hierarchical organization of certain regions or the mirror systems for action learning and imitation, has also been shown useful in different tasks.

One limitation of these types of approaches is that we are still far from a deep understanding of the functioning of the brain, and this makes the task of actually replicating its functionality virtually impossible. Even though we already understand many mechanisms in the brain that have shown good results when implemented in artificial systems, there are still many tasks where those systems are far from the high performance reached by the human brain. Another disadvantage when trying to mimic the brain is the fact that the human brain has about 86 billion neurons and 100 trillion connections between them, and current computers are still far from such high computational power. Therefore, hardware limitations may be prohibitive when trying to mimic the human brain in performing certain tasks. Still, note that models may be found that emulate the behavior of certain brain regions with an acceptable performance while having a considerably lower internal complexity. Finally, similar to classical machine learning and deep learning approaches, brain-inspired architectures are in general less expressive than logic-based ones, being hard to interpret how they have reached a particular conclusion. However, this depends on the particular framework used, and there are mechanisms that can alleviate this issue.

## 5. Discussion

The main aim of this review was to provide a general view on the problem of activity, plan, and goal recognition. This is a relevant problem in applications with humans in the loop, since the ability to recognize actions and intentions is a key element that allows us humans to interact naturally with others. Several reviews exist that describe, either the problem of activity recognition, or the problem of plan and goal recognition, together with their corresponding most common approaches. However, to the best of our knowledge, there are no reviews covering the whole problem of activity, plan, and goal recognition in a comprehensive way. The purpose of this review is precisely to fill this gap from the literature. To do so, we have started with a brief introduction to the mechanisms that are believed to take part in the process of action and intention recognition in humans, followed by a definition, possible taxonomy and main challenges of the problem and a description of the main approaches that have been proposed to address it.

As we have seen in section 3.2, there are many possible criteria to classify these systems, such as according to their objective (activity, plan, and/or goal recognition) or according to the type of approach followed to address the problem (logic-based, classical machine learning, deep learning, or brain-inspired). Other possible criteria attend to the characteristics of the environment and the agents. For example, real environments (e.g., in human-robot interaction applications) are generally characterized as being partially observable, stochastic and continuous. In the case they are also open environments, the recognition system will need to deal with unknown actions, plans, and goals. Table 1 shows a classification of the original studies that have been presented along this review according to the criteria described in section 3.2. These criteria may be used in future research studies to classify the presented approaches, allowing the community to better understand the scope of applicability and enabling a more straightforward comparison of approaches. In addition, they may also inspire the development of more standardized performance evaluation and comparison methods, which could consist, for instance, of a set of standard problems/datasets of different nature. These standard problems should allow us to better understand the scope of applicability of each approach, as well as their performance in the different types of applications.

**Table 1:** Classification of the original studies presented along the review according to their approach (logic-based, classical machine learning, deep learning, or brain-inspired), objective (activity/action, plan, or goal recognition), observed agent involvement (agnostic, adversarial, or intended), observer intervention (no intervention, offline intervention, online intervention, or direct communication), environment (fully or partially observable, deterministic or stochastic, and discrete or continuous), recognition time (offline or online), knowledge of possible classes, i.e., activities/plans/goals (known or unknown), number of agents (single or multiple), and application.

**Reference**	**Objective**	**Agent**	**Intervention**	**Environment**			**Recognition**	**Classes**	**#Agents**	**Application**
**Logic-based approaches**										
Shrager and Finin (1982)	Goal	Agnostic	No	Fully obs.	Det.	Disc.	Online	Known	Single	Software help
Avrahami-Zilberbrand and Kaminka (2014)	Plan	Adversarial	No	Part. obs.	Stoch.	Cont.	Online	Unknown	Single	Surveillance
Perrault and Allen (1980)	Goal	Intended	No	Fully obs.	Det.	Disc.	Offline	Known	Single	Language und.
Keren et al. (2014)	Goal	Agnostic	Offline	Fully obs.	Det.	Disc.	Online	Known	Single	Destin. guess
Shvo and McIlraith (2020)	Goal	Agnostic	Online	Part. obs.	Stoch.	Cont.	Online	Known	Single	General
Mirsky et al. (2018)	Plan	Intended	Direct	Fully obs.	Det.	Disc.	Online	Known	Single	Software help
Keren et al. (2016)	Goal	Agnostic	Offline	Part. obs.	Det.	Disc.	Online	Known	Single	Destin. guess
Wayllace et al. (2016)	Goal	Agnostic	Offline	Fully obs.	Stoch.	Disc.	Online	Known	Single	Destin. guess
Kaminka et al. (2018)	Plan	Agnostic	No	Part. obs.	Stoch.	Cont.	Online	Known	Single	General
Vered et al. (2016)	Goal	Agnostic	No	Fully obs.	Det.	Cont.	Online	Known	Single	Destin. guess
Zhuo (2014)	Plan	Agnostic	No	Part. obs.	Det.	Disc.	Offline	Known	Multiple	Theoretic
Genter et al. (2011)	Goal	Agnostic	No	Fully obs.	Det.	Disc.	Online	Known	Multiple	Video games
Skocir et al. (2016)	Activity	Agnostic	No	Part. obs.	Stoch.	Cont.	Online	Known	Single	Smart homes
Ha et al. (2014)	Goal	Agnostic	No	Fully obs.	Det.	Cont.	Online	Known	Single	Video games
Bouchard et al. (2007)	Plan	Agnostic	No	Part. obs.	Stoch.	Cont.	Online	Unknown	Single	Elderly care
(Sengupta et al., 2017)	Plan	Intended	Direct	Fully obs.	Det.	Disc.	Online	Unknown	Single	Decision support
Kautz and Allen (1986)	Plan	Agnostic	No	Fully obs.	Det.	Disc.	Offline	Known	Single	Theoretic
Zhuo (2017)	Plan	Agnostic	No	Fully obs.	Det.	Cont.	Offline	Known	Single	Experimental
Schank and Abelson (1977)	Plan	Intended	No	Fully obs.	Det.	Disc.	Offline	Known	Single	Language und.
Sohrabi et al. (2016)	Plan	Agnostic	No	Part. obs.	Det.	Disc.	Offline	Known	Single	Theoretic
Jarvis et al. (2004)	Plan	Adversarial	No	Part. obs.	Stoch.	Cont.	Online	Known	Single	Surveillance
Pereira and Han (2009)	Goal	Agnostic	No	Part. obs.	Stoch.	Cont.	Online	Known	Single	Elderly care
Raghavan et al. (2014)	Plan	Agnostic	No	Fully obs.	Det.	Disc.	Offline	Known	Single	Various
Quaresma and Lopes (1995)	Plan	Agnostic	No	Fully obs.	Det.	Disc.	Offline	Known	Double	Language und.
Vered and Kaminka (2017)	Goal	Agnostic	No	Part. obs.	Det.	Cont.	Online	Known	Single	Destin. guess
Myers (1997)	Plan	Intended	No	Part. obs.	Det.	Disc.	Offline	Known	Single	Planning help
Geib and Goldman (2009)	Plan	Agnostic	No	Part. obs.	Det.	Disc.	Online	Known	Single	Theoretic
Ramirez and Geffner (2009)	Plan	Agnostic	No	Fully obs.	Det.	Disc.	Online	Known	Single	Destin. guess
Pereira et al. (2020)	Goal	Agnostic	No	Part. obs.	Stoch.	Cont.	Online	Known	Single	General
Granada et al. (2017)	Action/Plan	Agnostic	No	Part. obs.	Stoch.	Cont.	Offline	Known	Single	Cooking
Amado et al. (2018b)	Action/Goal	Agnostic	No	Part. obs.	Det.	Disc.	Online	Known	Single	Puzzle solving
**Classical machine learning approaches**										
Akkaladevi and Heindl (2015)	Action	Intended	No	Part. obs.	Stoch.	Cont.	Online	Known	Single	Human-robot int.
Han and Pereira (2013)	Goal	Agnostic	No	Part. obs.	Stoch.	Cont.	Online	Known	Single	Elderly care
Kelley et al. (2010)	Action/Goal	Agnostic	No	Part. obs.	Stoch.	Cont.	Online	Known	Multiple	Human-robot int.
Saria and Mahadevan (2004)	Action/Plan	Agnostic	No	Part. obs.	Stoch.	Cont.	Online	Known	Multiple	Experimental
Laviers et al. (2009)	Goal	Adversarial	No	Fully obs.	Stoch.	Cont.	Online	Known	Multiple	Video games
Oh et al. (2014)	Plan	Intended	No	Fully obs.	Det.	Disc.	Online	Known	Single	Assist. agents
Horvitz et al. (1998)	Goal	Agnostic	No	Fully obs.	Det.	Disc.	Online	Known	Single	Software help
Rebelo et al. (2013)	Activity	Intended	No	Part. obs.	Stoch.	Cont.	Online	Known	Single	Orthotics
Liao et al. (2004)	Action/Goal	Agnostic	No	Part. obs.	Stoch.	Cont.	Online	Unknown	Single	Destin. guess
Oliver et al. (2002)	Activity	Agnostic	No	Part. obs.	Stoch.	Cont.	Online	Known	Single	Office awar.
Bui et al. (2004)	Action/Plan	Agnostic	No	Part. obs.	Stoch.	Cont.	Online	Known	Single	Surveillance
Zhao et al. (2010)	Activity	Agnostic	No	Part. obs.	Stoch.	Cont.	Offline	Known	Single	Experimental
Liao et al. (2007)	Activity	Agnostic	No	Part. obs.	Stoch.	Cont.	Offline	Known	Single	Daily life
Baker and Tenenbaum (2014)	Plan	Agnostic	No	Part. obs.	Stoch.	Cont.	Online	Known	Single	Experimental
Samanta and Chanda (2014)	Activity	Agnostic	No	Part. obs.	Stoch.	Cont.	Online	Known	Single	Video tagging
Fan et al. (2013)	Activity	Agnostic	No	Part. obs.	Stoch.	Cont.	Offline	Known	Single	Daily life
Vahdatpour et al. (2009)	Activity	Agnostic	No	Part. obs.	Stoch.	Cont.	Online	Unknown	Single	Elderly care
Polyvyanyy et al. (2020)	Goal	Agnostic	No	Part. obs.	Stoch.	Cont.	Online	Unknown	Single	General
**Deep learning approaches**										
Meng and Huang (2018)	Goal	Intended	No	Fully obs.	Det.	Disc.	Offline	Known	Single	Language und.
Rahmat et al. (2018)	Goal	Adversarial	No	Fully obs.	Det.	Disc.	Online	Unknown	Single	Net. security
Hammerla et al. (2016)	Activity	Agnostic	No	Part. obs.	Stoch.	Cont.	Online	Known	Single	Daily life
Bevilacqua et al. (2018)	Activity	Agnostic	No	Part. obs.	Stoch.	Cont.	Online	Known	Single	Experimental
Ronao and Cho (2016)	Activity	Agnostic	No	Part. obs.	Stoch.	Cont.	Online	Known	Single	Daily life
Amado et al. (2018a)	Goal	Agnostic	No	Part. obs.	Det.	Disc.	Offline	Known	Single	Experimental
Ordóñez and Roggen (2016)	Activity	Agnostic	No	Part. obs.	Stoch.	Cont.	Online	Known	Single	Daily life
Zhang et al. (2015)	Activity	Agnostic	No	Part. obs.	Stoch.	Cont.	Online	Known	Single	Daily life
Min et al. (2014)	Goal	Agnostic	No	Fully obs.	Det.	Cont.	Online	Known	Single	Video games
Sheng and Huber (2019)	Activity	Agnostic	No	Part. obs.	Stoch.	Cont.	Online	Known	Single	Daily life
Dwivedi et al. (2019)	Activity	Agnostic	No	Part. obs.	Stoch.	Cont.	Online	Known	Single	Video tagging
Xian et al. (2020)	Activity	Agnostic	No	Part. obs.	Stoch.	Cont.	Offline	Known	Multiple	Video tagging
**Brain-inspired approaches**										
Kuniyoshi et al. (2003)	Action	Agnostic	No	Part. obs.	Stoch.	Cont.	Online	Unknown	Single	Human-robot int.
Bonaiuto et al. (2005)	Action	Agnostic	No	Part. obs.	Stoch.	Cont.	Online	Known	Single	Experimental
Oztop et al. (2005)	Action/Goal	Adversarial	No	Part. obs.	Stoch.	Cont.	Online	Known	Single	Experimental
Haruno et al. (2001)	Action	Agnostic	No	Part. obs.	Stoch.	Cont.	Online	Unknown	Single	Human-robot int.
Demiris and Khadhouri (2005)	Action/Goal	Agnostic	No	Part. obs.	Stoch.	Cont.	Online	Known	Single	Human-robot int.
Yamashita and Tani (2008)	Action	Agnostic	No	Part. obs.	Stoch.	Cont.	Online	Known	Single	Human-robot int.
Alkurdi et al. (2018)	Action	Agnostic	No	Part. obs.	Stoch.	Cont.	Online	Known	Single	Daily life
Oltramari and Lebiere (2014)	Action	Agnostic	No	Part. obs.	Stoch.	Cont.	Online	Known	Single	Surveillance

Table 1, while not meant to be exhaustive, can also give an idea about what kinds of systems have been extensively studied and which ones are still not well-explored. For example, the table shows that most existing work considers agnostic actors, non-intervening observers, known possible activities/plans/goals and single agent systems. However, applications in real environments, for instance, may require a further exploration of scenarios with both adversarial and intended actors, with online intervention and/or direct communication, with unknown possible actions/plans/goals and with multiple agents.

On the other hand, very few of the surveyed systems do action and plan recognition at the same time. In fact, most logic-based approaches focus on plan or goal recognition, while the rest of the approaches are mainly used for activity or goal recognition. This is coherent with the characteristics of those approaches, which are summarized in Table 2. This table compares the four proposed types of approaches attending to some of their properties, as well as to their ability to deal with different challenges. This way, hybrid logic-probabilistic approaches are probably the most commonly used ones for plan and goal recognition due to their high expressivity and the possibility to define different kinds of relationships among entities, while at the same time being able to handle uncertainty. They have the limitation that they usually require a manual introduction of a relevant part of the necessary knowledge. This generally implies much domain-expert human effort and systems that are hard to generalize, bad at scaling, prone to errors, and unable to handle plans or goals out of the scope of their domain knowledge. Deep learning approaches, on the other hand, are the natural evolution of classical machine learning techniques, and are becoming the main solution to the problem of activity recognition, due to their ability to deal with raw sensor data, extract features at different abstraction levels, and learn very different patterns from the training data (e.g., intraclass variability, ambiguous activities, etc.). These approaches, however, usually require large amounts of labeled data, which, again, implies much human effort. In addition, they are not good at handling and learning new activities online. Finally, brain-inspired approaches are an alternative that is sometimes used when it comes to real environments where the recognition needs to be done online (e.g., in human-robot interaction). These algorithms are hard to evaluate and compare with the others due to their great diversity and to the fact that they have not been in general as extensively explored. However, they show characteristics that place them as promising candidates to solve, in the near future, some of the yet unsolved challenges present in real unconstrained environments (e.g., learning new actions, plans and goals in an unsupervised and online way, and in few shots, similarly to how we humans do).

**Table 2:** Summary comparison among the types of approaches in terms of their ability to represent structured data, their expressivity, their capacity to deal with uncertainty, their flexibility to adapt to different data, their robustness, their competency at handling sensor data, the human effort required to develop the system, their scalability, their aptness to deal with open environments, and the maturity of the technology.

**Properties/challenges**	**Logic-based**	**Classical machine learning**	**Deep learning**	**Brain-inspired**
Structure	+ +	−	+	+
Expressivity	+ +	o	−	o
Uncertainty	o	+ +	+ +	+ +
Flexibility	−−	+	+ +	+
Robustness	−	+	+ +	+
Sensory input	−−	o	+ +	+
Human effort	−−	−	−	o
Scalability	−	−	+	o
Open environment	−	−	o	+
Maturity	+ +	+ +	+	−

*A score is presented (− −, −, o, +, + +) for each type of approach and criterion, with the scores ranging from − − (very bad at it) to + + (very good at it)*.

While systems addressing the whole problem are not common in the literature, end-to-end systems that are able to recognize from primitive actions to high-level plans may be very useful and even necessary in different applications. One way to approach this problem may be to combine existing action recognition and plan recognition systems. For example, Granada et al. (2017) built a logic-based plan recognition system upon a deep learning action recognition system. However, the similarities between the two sub-problems may justify the development of more homogenous systems. Indeed, as commented in section 3.1, the three sub-problems present similar characteristics and need to deal with similar challenges, and the whole problem can be considered a hierarchical task in which the different levels of the hierarchy can be seen as performing action or goal/plan recognition with respect to the higher or lower levels, respectively. Some examples of existing systems approaching the whole problem in a unified way are those relying on hierarchical probabilistic models (e.g., Saria and Mahadevan, 2004). Another direction that could lead to successful unified solutions could be to find suitable ways of combining logic-based and deep learning approaches in a way that the advantages of both worlds can be exploited, similar to what was done with logic-based and probabilistic approaches (some work in this direction has already been done, e.g., see Amado et al., 2018b). These hybrid systems could be very good at dealing with sensory data while at the same time being very expressive at describing complete and complex plans. A further investigation of deep learning and brain-inspired approaches in the context of plan recognition may also lead to new insights. In particular, bringing ideas from the brain mechanisms involved in the human recognition of plans to the well-established deep-learning networks may contribute to new successful systems. Ideas from cognitive science and from our knowledge on disorders related to the recognition of intentions or actions may also inspire the development of new successful brain-inspired computational systems.

Concerning the applications, some commercially successful action, plan, and goal recognition systems already exist in areas as diverse as virtual assistants or fitness/activity tracking. However, even in these successful areas, the performance is often not up to the user expectations or demands. In addition, there are many areas where the transition of new technologies from academia to common-use real world applications, together with the expected increasing demand, may soon bring important breakthroughs, such as human-robot interaction or elderly care. On the other hand, considering the recent boost in the areas of computer vision and natural language understanding, we can also expect important advances in these areas in the following years. This way, applications relying on these technologies to perform action or goal recognition will also experience important advancements, as well as applications relying on technologies that can take advantage from advances in deep learning.

In conclusion, hybrid logic-probabilistic approaches and deep learning approaches are probably the ones that are showing better results as of today in the problems of plan/goal recognition and activity recognition, respectively. Nevertheless, they still require further research to improve their performance and to tackle the challenges that are not able to address properly yet (e.g., dealing with unknown activities, plans, or goals), as well as to be able to deal with the complete problem. On the other hand, brain-inspired approaches, while not so extensively explored, count on some promising characteristics, and a further development may help better address those challenges. This way, combining ideas from these different approaches may contribute to finding new ways to address the yet challenging issues, as well as to find end-to-end solutions to the whole problem.

## Author Contributions

FV-H did the literature research and analysis and the writing of the manuscript. Both authors contributed to the conceptualization, review, editing, and approval of the final version.

## Conflict of Interest

The authors declare that the research was conducted in the absence of any commercial or financial relationships that could be construed as a potential conflict of interest.
